# A Full-Range Flexible and Printed Humidity Sensor Based on a Solution-Processed P(VDF-TrFE)/Graphene-Flower Composite

**DOI:** 10.3390/nano11081915

**Published:** 2021-07-26

**Authors:** Shenawar Ali Khan, Muhammad Saqib, Muhammad Muqeet Rehman, Hafiz Mohammad Mutee Ur Rehman, Sheik Abdur Rahman, Yunsook Yang, Seongwan Kim, Woo-Young Kim

**Affiliations:** Department of Electronic Engineering, Jeju National University, 102 Jejudaehakro, Jeju 63243, Korea; shenawaralikhan@jejunu.ac.kr (S.A.K.); saqibmuhammad@jejunu.ac.kr (M.S.); muqeet1988@jejunu.ac.kr (M.M.R.); mutee1990@jejunu.ac.kr (H.M.M.U.R.); abdurrahman@jejunu.ac.kr (S.A.R.); yunsuk0001@jeunu.ac.kr (Y.Y.); pea8543@jejunu.ac.kr (S.K.)

**Keywords:** humidity sensor, P(VDF-TrFE)/graphene flower composite, flexible, solution processed, health monitoring

## Abstract

A novel composite based on a polymer (P(VDF-TrFE)) and a two-dimensional material (graphene flower) was proposed as the active layer of an interdigitated electrode (IDEs) based humidity sensor. Silver (Ag) IDEs were screen printed on a flexible polyethylene terephthalate (PET) substrate followed by spin coating the active layer of P(VDF-TrFE)/graphene flower on its surface. It was observed that this sensor responds to a wide relative humidity range (RH%) of 8–98% with a fast response and recovery time of 0.8 s and 2.5 s for the capacitance, respectively. The fabricated sensor displayed an inversely proportional response between capacitance and RH%, while a directly proportional relationship was observed between its impedance and RH%. P(VDF-TrFE)/graphene flower-based flexible humidity sensor exhibited high sensitivity with an average change of capacitance as 0.0558 pF/RH%. Stability of obtained results was monitored for two weeks without any considerable change in the original values, signifying its high reliability. Various chemical, morphological, and electrical characterizations were performed to comprehensively study the humidity-sensing behavior of this advanced composite. The fabricated sensor was successfully used for the applications of health monitoring and measuring the water content in the environment.

## 1. Introduction

Various environmental parameters have direct effects on human life, industrial production, and agricultural growth. These environmental parameters include light, temperature, pressure, and humidity. The monitoring of these parameters can produce significant results; the sensing of humidity levels, especially, has many applications, including those in the medical science, food processing, climate control, and semiconductor industries [1,2,3,4]. Investigating the behaviour of various materials as the functional layer of humidity sensors is among the most researched areas in recent times leading to several working mechanisms taking place inside these electronic devices. The best choice for sensing the response of humidity is the sensor based on change in values of capacitance or impedance with relative humidity [5,6,7]. Due to low cost, environmental friendliness, and ease of integration with electronic circuits, these sensors have been the subject of much interest. They are fabricated to detect water content levels in different environments. Recent research on humidity sensors has focused on improving the detection range, response and recovery time, reproducibility, fabrication methodology, cost and sensitivity level [8,9]. The responsivity of the sensor depends on the surface area of sensing material exposed to water content. Although different materials with humidity-sensing properties have been investigated, including metal oxides, carbon nanotubes, polymers, composites and semiconductor nanoparticles [10,11,12,13,14,15,16], new functional materials for sensing humidity responses are still required to further improve responsivity capabilities, sensitivity and reliability.

Among novel materials, two-dimensional (2D) nanomaterial structures are investigated widely due to their high surface areas. Graphene is the pioneer and most attractive 2D material for use in different applications, owing to its excellent thermal, mechanical, and electrical properties. The distinguished characteristics of graphene are due to its two-dimensional monolayer of carbon atoms forming a one-atom-thick honeycomb-like structure, making it useful for ultra-sensitive detection applications because of its large surface-to-volume ratio [17,18,19,20,21] Graphene-based gas sensors with the sensitivity as high as sensing the single gas molecule have been reported [22,23,24]. Due to their p-orbital electrons, graphene forms pi-bonds with surrounding atoms, and electrons of these pi-bonds are responsible for high sensitivity behaviour to any environmental change, making graphene ideal for use in chemical and biological sensing applications [19,25]. Sensors based on graphene as an active sensing layer have been reported for humidity sensing, but due to high conductivity of the graphene monolayer structure, the outcomes of those studies showed poor sensitivity to low humidity levels. To solve this problem, the sensitivity and detectable range of a graphene-based humidity sensor can be improved by blending it with some other material.

A second class of materials with high sensitivity for different environmental parameters is known as polymers. In addition, other advantages of using these materials include low cost, easy fabrication, excellent stability, and suitable mechanical properties for flexible electronics. Poly (vinylidene fluoride-trifluoroethylene) (P(VDF-TrFE)) belongs to this class of materials with excellent mechanical and ferroelectric properties that can be conformed to many complex surfaces among various polymers. In addition, it shows high thermal and chemical stability levels. This material has already shown promising results in pressure sensors, health monitoring devices, and temperature-sensing applications [26,27]. It has also been demonstrated that the dielectric properties of this polymer material can change upon a change in the relative humidity in the surrounding environment [28,29]. The addition of 2D materials nanoparticles to a polymer-based solution can improve the electrical properties of sensing devices [30].

In this work, for the first time, we demonstrate an ultra-fast, flexible, and highly sensitive humidity sensor based on novel sorption layer (multi-layered graphene nanostructures known as graphene flower and P(VDF-TrFE) composite) for potential applications in printable electronics. Graphene flower has transparent nature, exceptional conductivity, a highly crystalline structure, and large surface area of 500–2500 m^2^/g [31,32,33,34]. Thus, it can be concluded that graphene flower can be a suitable candidate for humidity sensing applications [35]. Interdigital electrodes (IDEs) were fabricated through screen printing technology, and an active sensing layer was deposited on these IDEs through spin-coating. The fabricated sensor exhibited quick response and recovery times of 0.8 s and 2.5 s, respectively. The proposed sensor exhibited high sensitivity of 0.0558 pF/% RH within a detectable range of (8–98)% RH and the excellent stability for 15 days. Furthermore, the fabricated humidity sensor was also tested for various real-time applications such as non-contact humidity sensing, mouth, and nasal breathing test. Hence, with these given aspects such as cost-effectiveness, flexibility, and high sensitivity, this proposed sensor is suitable for the wearable applications.

## 2. Experimental Methods

### 2.1. Materials and Methods

**Figure 1:**
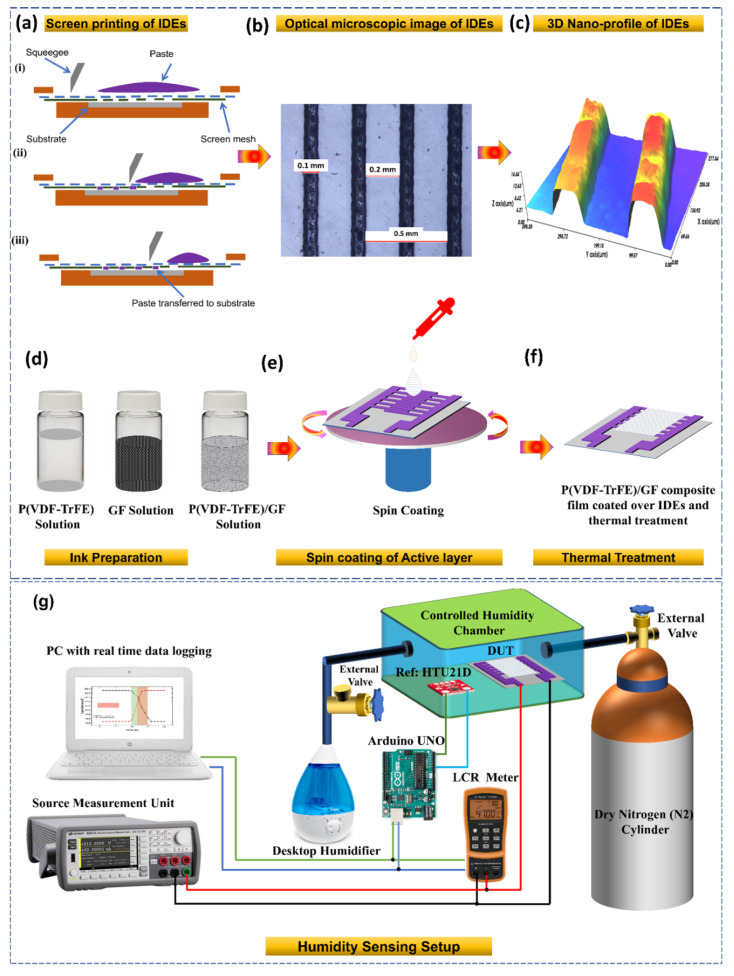
(**a**) Fabrication process of interdigital electrodes. (**b**) Optical microscopic image of IDEs. (**c**) 3D Nano-profile of IDEs. (**d**) Schematic diagram of the ink preparation of P(VDF-TrFE), GF solution and their composite. (**e**) Deposition of active layer through spin coating (**f**) Thermal treatment of the composite film at 80 °C. (**g**) Experimental setup used to characterize the humidity sensor.

Graphene flower (GF) solution in methyl-ethyl-ketone (MEK) (99.9 wt%) was purchased from InALA, Kobe, Japan. The N-methyl-2-pyrrolidone (NMP) solvent, which has a purity of 99.5%, was purchased from Dae-Jung Chemicals Ltd., Siheung-si, Gyeonggi-do, Korea. P(VDF-TrFE) polymer was purchased from Elf Atochem, North America (Philadelphia, PA, USA). The screen-printing Ag ink TEC-PA-051LV with viscosity 155,000 ± 15,000 cps, density ~2.8 ± 0.2 g/cm^3^, and metal content ~70 ± 2 wt% for the preparation of electrodes was purchased from InkTec, Ansan-si, Gyeonggi-do, Korea. The PET substrate with a thickness of 100 µm was purchased from AgIC paper. The P(VDF-TrFE) solution was prepared by mixing the P(VDF-TrFE) pellets in MEK as a solvent by 10 wt%/vol and then heated it at 80 °C with continuous stirring for 24 h. The GF solution contains graphene flower nanoflakes in MEK as a solvent. After that, the composite ink of P(VDF-TrFE) and GF was prepared by mixing them in NMP solvent. The prepared final solution was placed on a magnetic stirrer for 30 min followed by ultrasonication to generate a homogeneous solution as illustrated in Figure 1d.

### 2.2. Sensor Fabrication

The detailed fabrication process of proposed humidity sensor is illustrated in Figure 1. Initially, the PET substrate was washed with ethanol and deionized water followed by drying at room temperature. The detailed fabrication process for patterning IDEs through screen printing (Automax System Engineering AMX-1240M) is illustrated in Figure 1a. The width of the electrode fingers was 100 µm, while spacing between two IDEs was 200 µm. After the fabrication of electrodes, the active layer was spin-coated onto the electrodes and subsequently annealed at 120 °C for 30 min in an open air environment to crystallize the thin layer of composite solution, as illustrated in Figure 1e,f. Furthermore, optical microscope and 3D nano-profile images of a patterned IDE shown in Figure 1b,c, indicate that the electrodes were properly fabricated on the surface of PET substrate with the thickness of 5 µm without any significant deformity.

### 2.3. Characterization

The surface morphology of the deposited active layer was investigated by the scanning electron microscope TESCON MIRA 3, while the elemental composition was explored using dispersive X-ray spectroscopy (EDS). The surface roughness and film thickness were examined by using the NV2000 three-dimensional optical surface profiler. The X-ray diffractometer (Panalytical X’PERT PRO) was utilized to characterize the crystal structure of GF and its composite with P(VDF-TrFE).

### 2.4. Sensor Evaluation

To measure the humidity-sensing performance of both sensors, we investigated the electrical properties under different levels of humidity. The experimental setup used for sensing the humidity is shown in Figure 1g. To achieve different levels of humidity, we used a custom-made controlled humidity environmental box. This humidity sensing setup made in-house had the ability to control its relative humidity levels from 0% RH to 100% RH. Dry nitrogen (N_2_) gas was used to decrease the level of humidity inside our measurement chamber; however, the desktop humidifier was used to supply water vapours inside the chamber to increase the level of RH%. All inlets were controlled electronically except for the dry N_2_ gas, which was manually operated using a mechanical valve. To calibrate the humidity levels in the chamber, a commercially available humidity sensor (HTU21D) was used along with an Arduino board and a digital display. The fabricated sensor was placed side by side with the commercial humidity sensor in the sealed measurement chamber for recording data. We recorded digital parameters, in this case the capacitance, impedance responses and the device current through an Keysight LCR meter U1733C and Keysight Source Measurement Unit B2911A. The source alternating voltage signal provided by LCR meter is 0.74 Vrms for the measurement of both capacitance and impedance response. Upon a change in the relative humidity of the chamber, the capacitance and impedance spectra containing the impedance and capacitance responses were obtained by interfacing the LCR meter with a computer system. The humidity responses for the proposed sensor were recorded for a broad humidity range and the temperature was maintained at 22 °C for the duration of the experiment. While measuring the electrical response of the sensor, the test frequency was kept constant at 10 kHz and 100 kHz. While recording the measurements, the humidification level of the chamber was gradually increased to obtain maximum data points.

## 3. Results and Discussions

**Figure 2:**
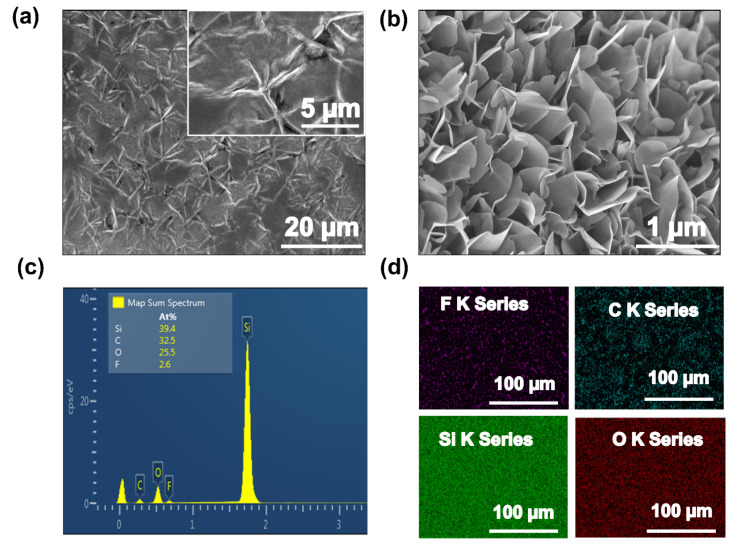

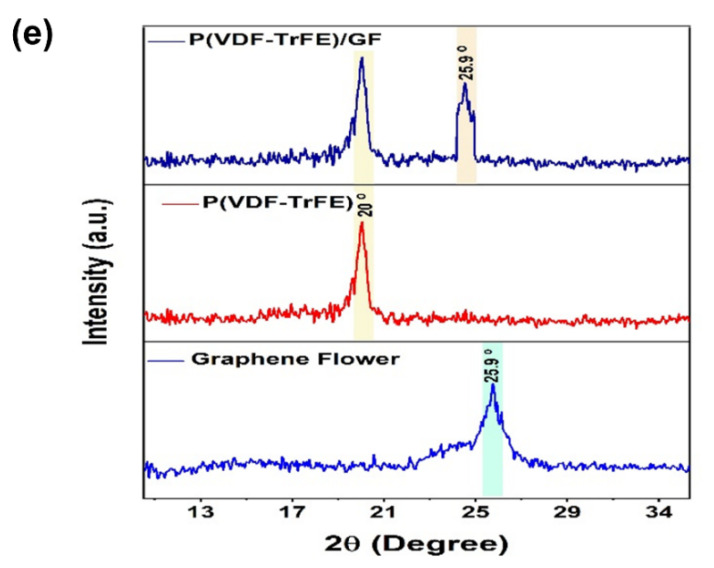
(**a**) SEM image of the deposited composite film P(VDF-TrFE)/GF composite film. (**b**) SEM image of GF. (**c**) EDS analysis of composite film showing the Si, C, O, and F elements in the active layer. (**d**) F K series, C K series, O K series, and Si K series. (**e**) XRD pattern of P(VDF-TrFE), GF, and their composite.

To visualize the surface morphology of P(VDF-TrFE)/GF composite thin film of the proposed humidity sensor, a SEM image of the active layer was taken with the magnification level of 20 µm, as illustrated in Figure 2a, which indicated the uniform dispersion of active material in the form of thin film onto the substrate, while the SEM image of pristine graphene flower with the magnification level of 1 µm is illustrated in Figure 2b. The elemental composition of the sensing layer was investigated by energy dispersive X-Ray spectroscopy EDS, with the magnification level of 100 µm. The layered EDS image demonstrates the presence of carbon (32.5)%, oxygen (25.5)%, fluorine series (2.6)% and silicon (39.4)%, respectively, as illustrated in Figure 2c. The investigated results confirm the presence of F K series, C K series, Si K series, and O K series, as shown in Figure 2d. This EDS mapping confirmed the presence of GF and P(VDF-TrFE) copolymer. XRD analysis was performed to investigate the crystalline structure of P(VDF-TrFE), GF and P(VDF-TrFE)/GF composite thin films using the X-ray diffractometer (Empyrean) at a step size of 0.02° from 10° to 60°. XRD analysis of P(VDF-TrFE)/GF composite thin film exhibited the peaks at 20° and 25.9°; as illustrated in Figure 2e, the diffraction peak at 2θ = 20° was attributed to the (110) and (200) orientation planes, which were associated with the polar β-phase and 25.9° (around 2θ = 27°), indicating that the product had carbon-based material composition and internal carbon atoms or a molecular structure.

**Figure 3:**
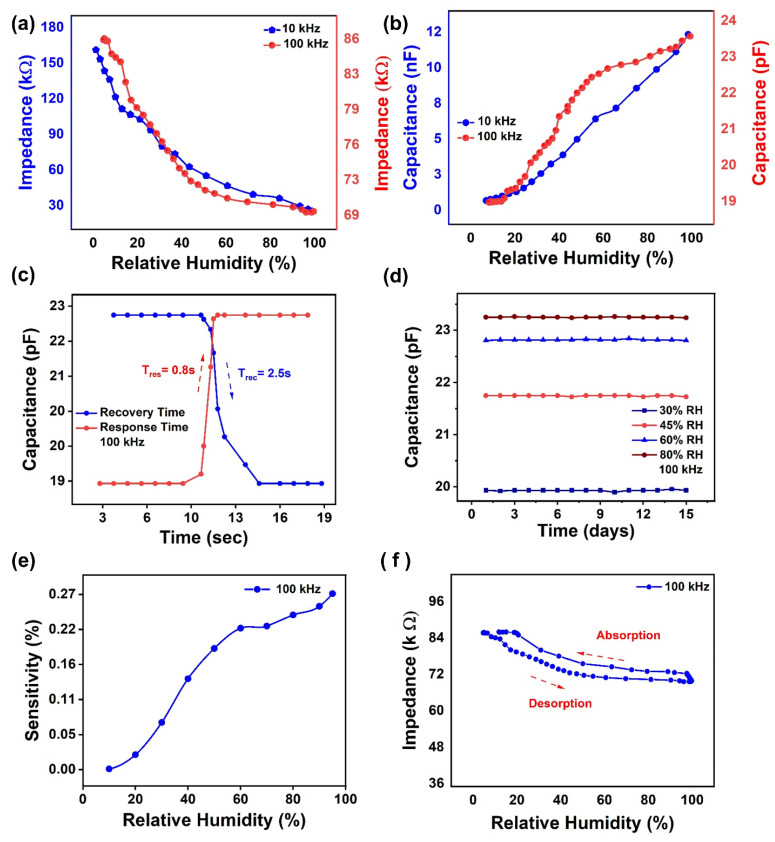
(**a**) Impedance response of the proposed humidity sensor at 10 kHz and 100 kHz. (**b**) Capacitance response of the proposed humidity sensor at 10 kHz and 100 kHz. (**c**) Capacitance response and recovery time of proposed humidity sensor at 10 kHz. (**d**) Capacitance stability of proposed humidity sensor over 15 days. (**e**) Capacitance sensitivity of proposed humidity sensor. (**f**) Hysteresis of the proposed sensor during absorption and desorption.

The electrical response of the fabricated humidity sensor was measured as the change of impedance and capacitance against the relative humidity at the test frequency of 10 kHz and 100 kHz. The measured impedance and capacitance responses were illustrated in Figure 3a,b. The measured result clearly shows that the impedance of the sensor decreased with an increase in the relative humidity. The investigated results depict that the impedance at the test frequency of 10 kHz starts at 170 kΩ at 8% RH and then decreases up to 26 kΩ, until 98% RH. Similarly, the most significant impedance variation of 86 kΩ–69 kΩ was observed at 8% RH to 98% RH at the test frequency of 100 kHz.

Similarly, capacitance response of the proposed humidity sensor was also recorded against the change in relative humidity at the test frequency of 10 kHz and 100 kHz, respectively. With an increase in relative humidity, absorption of water molecules increases into the surface of the active sensing layer, leading to a change in dielectric coefficient and therefore, changing the capacitance response of the sensor. It was observed that with an increase in relative humidity (8–98%) RH, the capacitance increased from 673 pF to 12 nF at the test frequency of 10 k Hz, as shown in Figure 3b. Apart from that, the capacitance response increase from 18 pF to 23 pF with the increase in relative humidity from (8–98%) RH, as illustrated in Figure 3b. The equivalent electrical circuit of a thin-film humidity sensor is shown in Figure S1 of the Supplementary Information. Each element of this circuit in Figure S1 represents a RC-transmission line. With the increase in relative humidity, the distributed resistance f this two-terminal device drops, and the capacitance changes. In addition, this value of capacitance is dependent on frequency, therefore requiring high frequency for large range of humidity detection [36].

Similarly, the transient response results are shown in Figure 3c. Notable response and recovery times of the sensor were determined using the transient response. The values of the sensor were measured with a commercially available humidity sensor (HTU21D) while changing the humidity levels from 8% RH to 98% RH. Response time is the time required by the sensor to reach a high humidity level from a low humidity level, and vice versa for the recovery time. The proposed humidity sensor had a stable transient response with a response time of 0.8 s and a recovery time of 2.5 s for capacitance. Similarly, the response and recovery times of the proposed sensor at the 10 kHz are illustrated in Supplementary Figure S2. Both values were highly desirable in applications requiring fast recovery and response times. A comparison of the sensing parameters of our humidity sensor with other previously reported sensors is illustrated in Table 1. It is clear from Table 1 that our proposed device showed remarkable sensing parameters and ultra-fast transient response, compared to other reported humidity sensors. Another key parameter to evaluate the performance of any sensor is its stability; therefore, we performed the stability test of our fabricated humidity sensor by placing it into certain fixed humidity environments, i.e., 30%, 60%, 72% and 80% RH levels for 15 days and observed capacitance of the sensor at the test frequency of 100 kHz. Figure 3d shows that the proposed sensor is highly stable at each RH level. Apart from that, the sensitivity of the sensor was calculated using the given mathematical Equation (1).

(1) $$S=\frac{C_{\mathrm{RH}}-C_{\mathrm{RH}o}}{C_{\mathrm{RH}o}}\times 100\%$$

where C_RH_ and C_RH__o_are the capacitances at the RH% and 8% RH. Figure 3e shows the relationship between sensitivity and capacitance of the sensor at the frequency of 100 kHz. From Figure 3e, it can be concluded that sensitivity of the sensor increases linearly from 0.00% to 0.27% with the average change of 0.0558 pF/% RH, which shows that the proposed sensor is highly sensitive to the relative humidity. Furthermore, the sensitivity of the proposed sensor at 10 kHz increases linearly from 0% to 14%, as illustrated in Supplementary Figure S3. Similarly, the hysteresis of the proposed sensor during absorption and desorption is illustrated in Figure 3f.

The dominant humidity-sensing mechanism can be explained by proton hopping, hydronium ion diffusion and by the Grotthuss mechanism [37,38]. At different humidity levels, water molecules form contacts with the P(VDF-TrFE)/GF composite layer. At a low humidity level, the probability of contact between the water molecules and the composite layer is low, as the water molecules do not form a continuous water layer. Thus, the transfer of water and hydronium ions on this discontinuous water layer becomes difficult. Therefore, this polymer composite layer showed high impedance at low humidity. When we increased the level of humidity, the number of water molecules absorbed in the nanostructured particles of graphene increased, resulting in an accelerated transfer of water and hydronium ions. This mechanism of ion transfer was presented by Grotthuss [39,40], and as this transfer of ions increases, impedance value decreases with an increase in humidity. In the working mechanism of the proposed device, water molecules cause the impedance to change in the composite film, which is then determined through the multiple fingers of IDEs to measure sensitivity.

The decrease in the values of impedance can also be explained by the electronic conduction occurring due to increase in water content within the composite film. This fabricated sensing device has very high impedance, and this impedance can decrease if the graphene nanoparticles are electrically connected to each other in any way, such as through the absorption of water vapor. Hence, the polymer-blended GF play a key role in creating electrical interconnections, and the absence of water molecules will result in an increase in the impedance. Thus, when water molecules reach the surface of sensing layer, they become absorbed in it, creating an electrical connection between the nanoparticles of GF, and decreasing the sample resistance, Rs.

**Figure 4:**
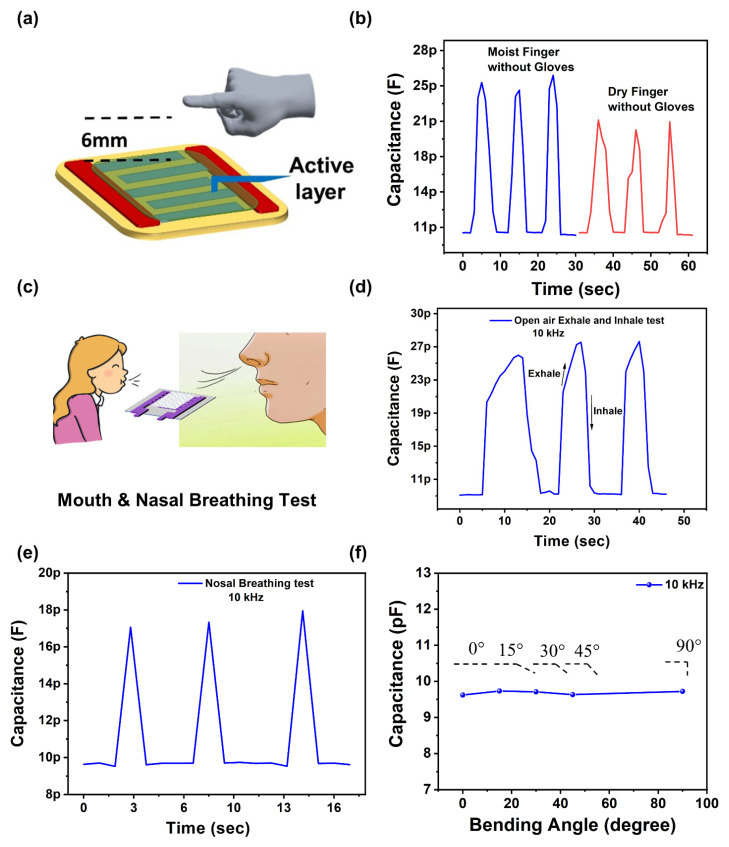
(**a**) Schematic diagram of contactless humidity sensing test of proposed sensor. (**b**) Capacitance response of moist finger and dry finger without gloves for contactless sensing experiments. (**c**) Schematic diagram Mouth and Nasal Breathing test of proposed sensor. (**d**) Schematic diagram of mouth breathing test of proposed sensor. (**e**) Capacitance curves of open-air exhale and inhale test. (**f**) Capacitance of the sensor when it is bended at different angles.

Based on remarkable sensing performance, the proposed sensor was tested for various applications such as non-contact humidity sensing of fingertip, mouth, and nasal breathing test. Schematic diagram of the non-contact humidity sensing for moist human fingertip with and without gloves at the distance of 6 mm is illustrated in Figure 4a. The capacitance of moist human finger without gloves increased from 9.9 pF to 24.3 pF, as shown in Figure 4b. Furthermore, we performed the same experiment by placing the dry finger at a distance of 6 mm from the sensor with the change in capacitance from 9.8 pF–21.3 pF at the frequency of 10 kHz. It can be concluded from the above results that our proposed sensor only responds to humidity without any insignificant error. Similarly, we also tested our device for a human health monitoring application by exposing this device to human breathing so that it can sense the water vapours. The schematic diagram of mouth breathing detection is illustrated in Figure 4c. Our device demonstrated a fast response time and recovery time with the capability to sense small RH% changes. Figure 4d shows the recorded data points for open-air exhale test and open-air inhale test of our proposed sensor with respect to normal breathing. While inhaling and exhaling the air, water vapours cause the change in the conductivity of the sensing layer. The measured change in capacitance is in picofarad range, therefore showing its promising potential in health monitoring application. Furthermore, we have performed the nasal breathing detection of our sensor by the keeping the distance between sensor and nose as 10 mm. From Figure 4e, it can be concluded that our sensor can perform nose breathing detection.

Flexibility also plays an important role in wearable applications. Finally, we performed the bending test of our sensor under various mechanical deformations, as illustrated in Figure 4f. The capacitance of the sensor was measured at different bending angles with test frequency of 10 kHz at the room temperature. The depicted results indicate some minor changes in capacitance, meaning that our sensor has the potential ability for wearable applications.

## 4. Conclusions

In this work, a highly stable, nearly linear, and flexible humidity sensor based on a novel sorption sensing layer (GF and P(VDF-TrFE) composite) with silver interdigitated electrodes printed through screen-printing was demonstrated. Physical, electrical, and chemical characterizations of the sensor were performed successfully. Graphene flowers were added to modify the electrical conduction behaviour of the polymer film, which is an important factor for humidity sensors. The proposed humidity sensor had a wide detection range of 8% RH–98% RH with a high sensitivity of 0.0558 pF/RH%. It also showed an ultra-fast transient response of 0.8 s and 2.5 s for the capacitance, with good stability for 15 days at various humidity levels. Based on its excellent sensing performance, we further demonstrated its performance for various applications such as non-contact detection, mouth, and nasal breathing detection. Similarly, the flexibility test of our proposed sensor was also performed. The fabrication process of the device is very simple, as it is printed on a low-cost flexible transparent PET substrate. This device has high potential to be integrated easily with wearable electronics.

## Figures and Tables

**Table 1:** A comparison table of the proposed sensor with other previously reported humidity sensors.

Sensing Material	RH Range	Response Time (s)	Recovery Time (s)	Linearity	Sensing Principle	Ref.
Graphene Oxide	10–98	19	10	-----	Piezoelectric	[41]
Reduced Graphene Oxide	30–90	28	48	Yes	Resistive	[42]
Tin(IV) Oxide/Reduced Graphene Oxide	11–97	102	6	------	-------	[43]
Chitosan/Graphene Quantum Dots	11–95	36	3	No	-----	[44]
Black Phosphorous	11–97	255	10	------	Resistive	[45]
Graphene Oxide/polyelectrolyte	11–97	----	----	No	Capacitive	[46]
Graphene-Polystyrene Sulfonic Sodium	30–95	3	22	No	Impedance Based	[47]
${\mathrm{VS}}_{2}$	0–100	30–40	12–50	No	Resistive	[48]
P(VDF-TrFE)/GF	8–98	0.8	2.5	No	Impedance Based	Our work

## Data Availability

The data presented in this study are available on request from the corresponding author.

## Supplementary Materials

The following are available online at https://www.mdpi.com/article/10.3390/nano11081915/s1, Figure S1: (a) The equivalent electrical circuit of a thin-film humidity sensor. (b) Cross Section of the sensor, Figure S2: Response and recovery times of the proposed sensor at 10 kHz, Figure S3: Sensitivity of the proposed sensor at 10 kHz.
