# Supplementary material for: A Full-Range Flexible and Printed Humidity Sensor Based on a Solution-Processed P(VDF-TrFE)/Graphene-Flower Composite
Source: Nanomaterials (Basel). 2021 Jul 26;11(8):1915. doi: 10.3390/nano11081915 (PMC8398072; doi:10.3390/nano11081915)
Supplement: Supplementary file 1 [file nanomaterials-11-01915-s001.zip › nanomaterials-1275428-supplementary.pdf]

Supplementary Materials

A Full-Range Flexible and Printed Humidity Sensor Based on a Solution-Processed P(VDF-TrFE)/Graphene-Flower Composite

Shenawar Ali Khan, Muhammad Saqib, Muhammad Muqeet Rehman, Hafiz Mohammad Mutee Ur Rehman, Sheik Abdur Rahman, Yunsook Yang, Seongwan Kim and Woo-Young Kim *

Department of Electronic Engineering, Jeju National University, 102 Jejudaehakro, Jeju 63243, Korea;
shenawaralikhan@jejunu.ac.kr (S.A.K.); saqibmuhammad@jejunu.ac.kr (M.S.); muqeet1988@jejunu.ac.kr (M.M.R.); mutee1990@jejunu.ac.kr (H.M.M.U.R.); abdurrahman@jejunu.ac.kr (S.A.R.); yunsuk0001@jeunu.ac.kr (Y.Y.); pea8543@jejunu.ac.kr (S.K.)
* Correspondence: semigumi@jejunu.ac.kr

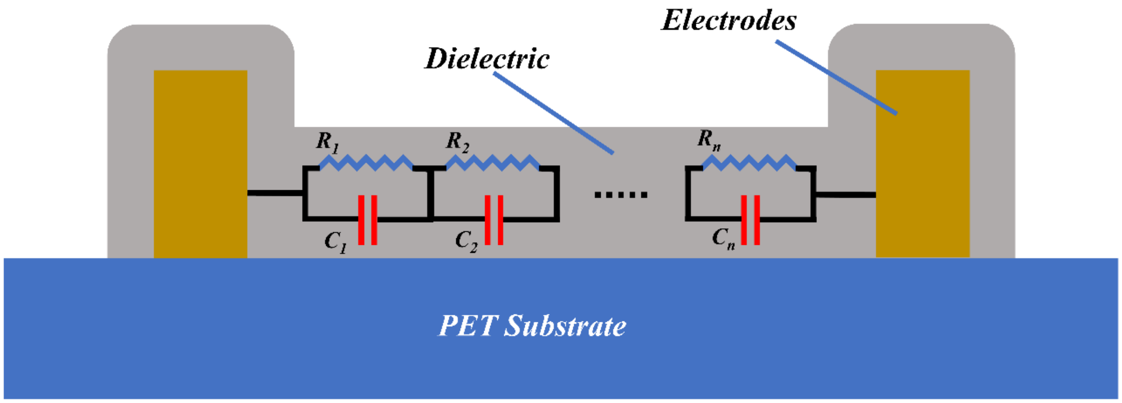

Figure S1. (a) The equivalent electrical circuit of a thin-film humidity sensor. (b) Cross section of the sensor.

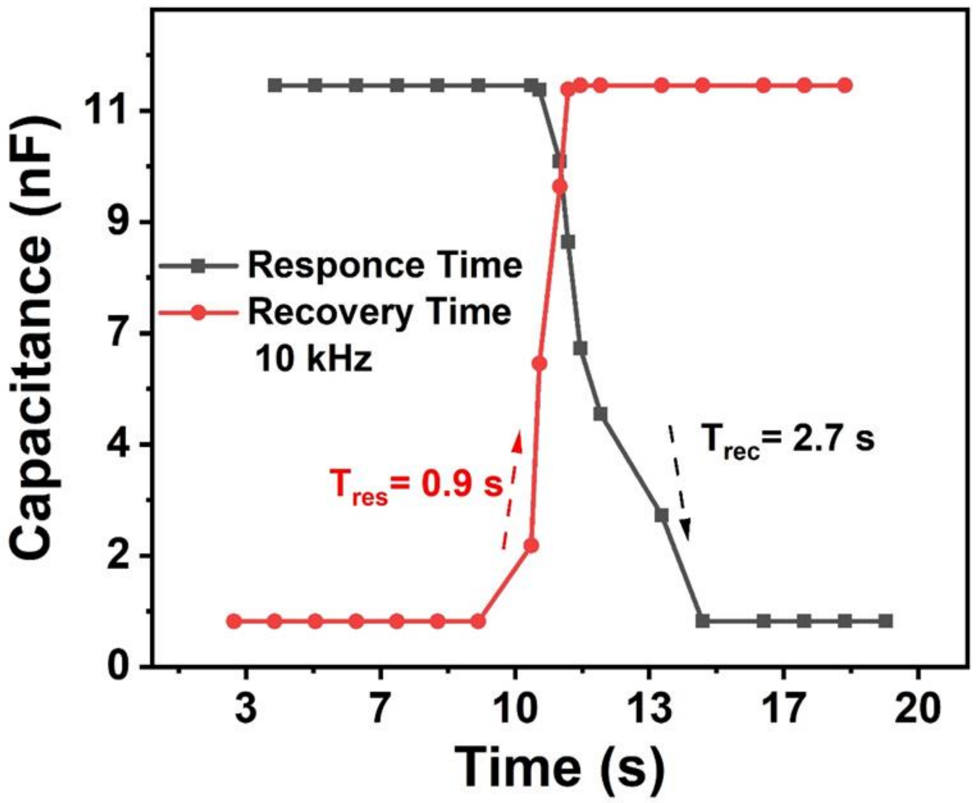

Figure S2. Response and recovery times of the proposed sensor at 10 kHz.

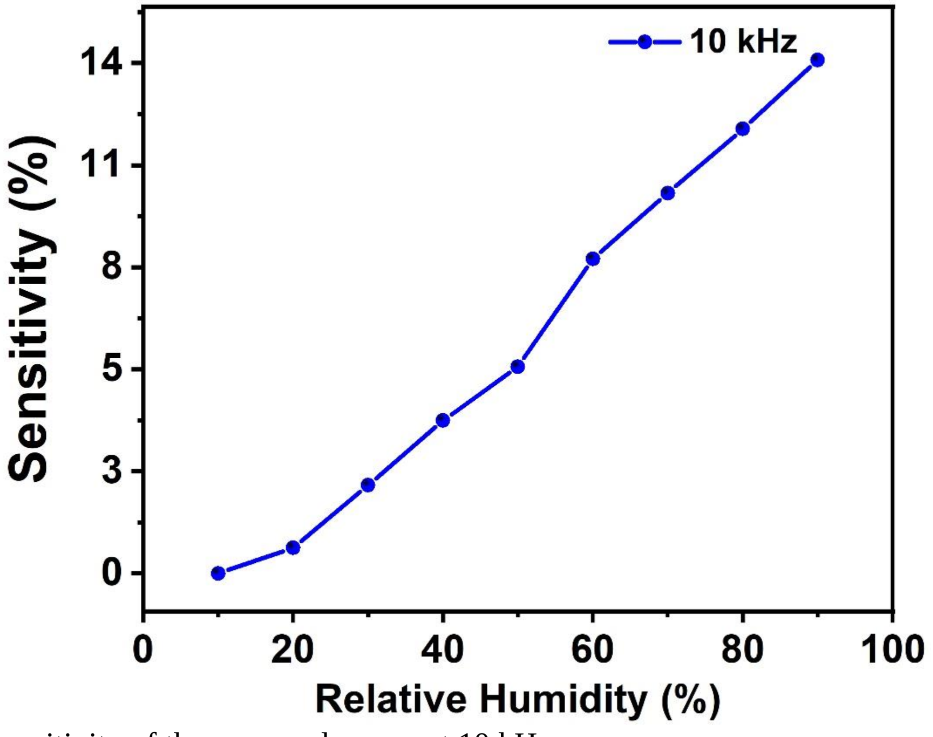

Figure S3. Sensitivity of the proposed sensor at 10 kHz.
